# Duration of Cochlear Microphonics in Click and Toneburst-Evoked Auditory Brainstem Response in Individuals With Auditory Neuropathy Spectrum Disorder and Normal Hearing

**DOI:** 10.7759/cureus.46734

**Published:** 2023-10-09

**Authors:** Megha Sasidharan, Madhuri Gore, Alex Mathew, Mary Praisy

**Affiliations:** 1 Department of Hearing Studies, Dr. S. R. Chandrasekhar Institute of Speech and Hearing, Bengaluru, IND

**Keywords:** normal hearing, clicks, tonebursts, auditory brainstem evoked response, cochlear microphonics, auditory neuropathy spectrum disorder

## Abstract

The presence of ringing cochlear microphonics (CM) with an absence of auditory brainstem response (ABR) is an indicator of auditory neuropathy spectrum disorder (ANSD). The duration of CM may vary based on the stimuli used to elicit the response. Generally, ABR is recorded using clicks with very limited use of tonebursts. Thus, this study aims to understand the duration of CM in individuals with ANSD and normal hearing in response to clicks, 500 Hz toneburst, and 4000 Hz toneburst using ABR. Results show that individuals with ANSD have a longer duration of CM than those with normal hearing. The presence of CM was more evident in response to toneburst stimuli than clicks, with 500 Hz being commonly eliciting more CM in both groups. The difference in duration of CM was statistically significant in individuals with ANSD with longer duration obtained for 500 Hz followed by clicks and 4000 Hz toneburst. The duration of the stimuli used plays an important role in revealing the CM while recording ABR. This indicates that the use of toneburst, particularly low frequency such as 500 Hz, will be clinically useful in identifying ANSD especially when otoacoustic emissions are compromised.

## Introduction

Cochlear microphonics (CM) is a preneural potential generated predominantly from the outer hair cells owing to their large number as compared to the inner hair cells [1]. The presence of CM with the absence of auditory brainstem response (ABR) is a classic feature of auditory neuropathy spectrum disorder (ANSD). There is evidence of ringing CM lasting from 4 to 6 ms in individuals with ANSD [2]. Thus, while testing young children with absent ABR where otoacoustic emissions are not recordable due to high noise floor or conductive hearing loss, one must always search for a CM [3]. The properties of CM are extensively studied using electrocochleography (ECochG) rather than ABR, owing to a better signal-to-noise ratio in the former [4]. It is almost always detected through the near field transtympanic ECochG even when the number of outer hair cells is compromised such as in those with profound hearing loss [5]. On the other hand, it is not always detected using scalp recordings in ears with sensorineural hearing loss [6]. This makes scalp recordings less useful to understand CM, although, the clinical relevance of CM in detecting ANSD is usually explored by ABR than ECochG. Studies on the comparison of CM in normal hearing versus ANSD are limited. Those available are mostly using ECochG [7]. In a study by Santarelli et al., ECochG results showed long-lasting CM in individuals with normal hearing with amplitudes comparable to ANSD [7]. They also found enhanced amplitude and duration of CM in individuals with central nervous system pathology, which could be attributed to the dysfunction of the efferent system. Thus, they concluded that CM may not be a distinctive feature of ANSD and a need to study the parameters of CM in individuals with ANSD and normal hearing. The studies on scalp-recorded CM obtained through ABR are seldom and have investigated predominantly the amplitude of the CM but not the duration. It is explored only using click stimuli in individuals with normal hearing versus individuals with ANSD [8]. Zhang [9], on the other hand, has assessed CM in response to different stimuli, i.e., clicks and tonebursts (short and long duration), in normal hearing but through ECochG and not scalp-recorded ABR.

Need for the study

The ABR can be misinterpreted as present in individuals with ANSD when in fact a cochlear microphonics is masquerading as a neural response [10], and sometimes in individuals with normal hearing, a neural response to low-frequency tonebursts may look like CM reversing in polarity with change in stimulus polarity [11]. An understanding of CM in normal hearing individuals versus ANSD is limited [12], and literature predominantly focuses on ECochG rather than scalp-evoked ABR to describe the CM, although ABR is more clinically used than ECochG. A clearer understanding of scalp-recorded CM in normal hearing versus ANSD could be useful in the differential diagnosis between profound hearing loss where peaks are absent, from similar neural impairments such as retrocochlear pathologies or delayed auditory maturation. Additionally, clicks are the most commonly used stimuli in clinical diagnosis as compared to toneburst stimuli. There is a need to study the behavior of toneburst-evoked ABR, especially in individuals with ANSD.

Aim

The study aims to describe and compare the duration of CM in individuals with ANSD and normal hearing using clicks, 500 Hz, and 4000 Hz toneburst ABR.

## Materials and methods

The data were collected retrospectively from 30 adults with ANSD (Group A) and 15 normal-hearing adults (Group B). No details disclosing patients' identity were used and this retrospective study is not funded by the institution or any other agency. The participants in Group A were diagnosed by experienced audiologists at the Department of Hearing Studies, Dr. S.R. Chandrasekhar Institute of Speech and Hearing, Bangalore as per the criteria given by Berlin et al. [13], with normal otoacoustic emissions (OAEs) and/or normal CM with absent or abnormal auditory brainstem response. The participants in Group B had normal hearing in pure tone audiometry (PTA) from 250 Hz to 8000 Hz within 15 dBHL and type A tympanogram with normal acoustic reflexes at 1000 Hz. The status of hearing in both the groups was based on case history, PTA using GSI 61 (Grason-Stadler, Eden Prairie, MN), and immittance using GSI TympStar (Grason-Stadler, Eden Prairie, MN). Group A had undergone the test battery, including OAEs using ILO V 6 (Otodynamics Ltd, Hatfield, UK). ABR was recorded using Bio-Logic Navigator Pro (Natus Medical Inc, Middleton, WI) in both the groups at 90 dBnHL using clicks (100 microseconds) and tonebursts of 500 Hz (10 ms) and 4000 Hz (1.25 ms) of 2-1-2 cycle duration, using a 10 ms time window. In Group B, the presentation level was lowered to 80 dBnHL wherever participants complained of discomfort. The waveform obtained in response to the rarefaction stimulus was superimposed on the waveform obtained in response to the condensation stimulus, to visualize the reversal of the CM phase with the change in stimulus polarity. The sensitivity levels were changed from 0.8 microvolts to 0.5 microvolts or to 0.2 microvolts when CM was too small to detect. To differentiate CM from the stimulus artifact, a third recording was made using rarefaction stimuli with the insert tubing pressed. If the ringing disappeared with tube press, it was considered as CM. In Group B, the CM was separated from peak I by subtracting the rarefaction waveform from the condensation waveform. This would enhance the CM and flatten the neural potentials as they would be in the same phase. In the ABR recorded in Group B, the duration of CM was obtained using the cursor at the point where the peaks ended, and no more reversing pattern was observed before peak I. On the other hand, the duration of CM in the retrospective data of ANSD reports was identified by scaling the time window into millimeters and converting it into milliseconds. The point where the reversing peaks flattened was marked and the time in milliseconds was calculated. In cases with a dilemma in identifying where the CM ended, a blind judgment was made by two experienced audiologists and two undergraduate students. A consensus between two or more similar judgments was considered for the analysis.

Statistical analysis

IBM SPSS version 20.0 (IBM Corp., Armonk, NY) was used for data analysis. The normality check was done using the Shapiro-Wilk test and non-parametric tests were applied. Within-group comparison for the type of stimuli used was done using the Kruskal-Wallis test and between-group comparison was made using the Mann-Whitney U test. A p-value < 0.05 was adopted for statistical significance.

## Results

**Figure 1:**
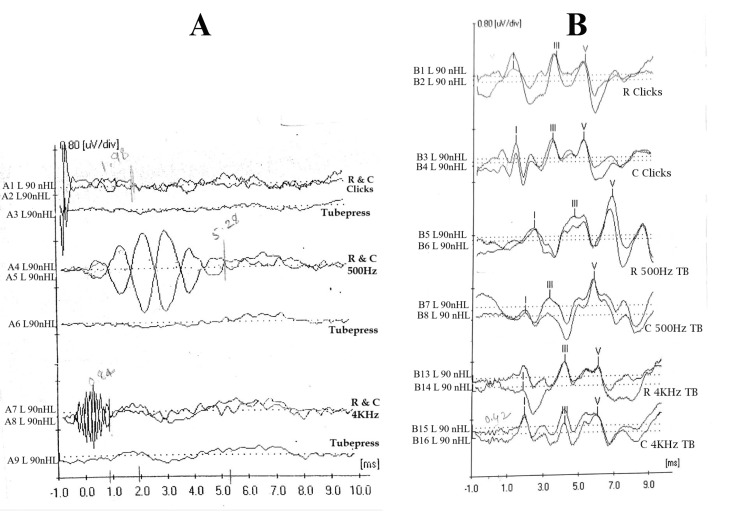
(A) ABR of an individual with ANSD. (B) ABR of an individual with normal hearing. ABR: auditory brainstem response; ANSD: auditory neuropathy spectrum disorder; R: rarefaction; C: condensation; L: left; A1 to A9 & B1 to B16: recording numbers; µv/div: microvolt per division; TB: toneburst; ms: millisecond; nHL: normalized hearing level.

The data with ABR recordings with artifacts were excluded from the study. Out of the ABR recordings collected from 30 individuals in Group A, 26 individuals with ANSD from the retrospective data were considered for the study. From these 26 individuals (52 ears), the data were further limited to 46 ears. The remaining data were excluded due to incomplete test protocol or test results. From Group B, three individuals were removed as the recording was poor due to electrical artifacts. Thus, the data were limited to 24 ears from Group B. Figure 1 shows the ABR recorded from an individual with ANSD and with normal hearing in response to clicks, 500 Hz toneburst, and 4000 Hz toneburst using rarefaction and condensation polarity. Tubepress or clamping of the insert tube was carried out to confirm the presence of CM.

The incidence rate of visible CM was more in Group A than in Group B (Table 1) and was longer in duration in Group A than in Group B. Tables 2, 3 show the descriptive statistics for Group A and Group B, with the mean and standard deviation for the duration of CM for three stimuli, i.e., clicks, 500 Hz toneburst, and 4000 Hz toneburst. In Group A, CM was present with a minimum duration of 0.84 ms and a maximum of 7 ms in response to clicks stimuli and Group B had CM present only in one ear with a duration of 1.07 ms. Similarly, in response to 500 Hz toneburst, in Group A, CM was present with a minimum duration of 2.49 ms and a maximum duration lasted throughout the time window of 10 ms. In Group B, CM had a minimum duration of 0.05 ms and a maximum of 1.2 ms. In response to 4000 Hz, in Group A, CM presented a minimum duration of 0.42 ms and a maximum of 3.1 ms. In Group B, CM presented with a minimum of 0.28 ms and a maximum of 1.03 ms duration. Thus, the presence of CM was more in response to toneburst stimuli than click stimuli in both groups. In Group A, the incidence of CM in response to both 500 Hz and 4000 Hz was similar but larger than clicks. On the other hand, in Group B, CM was clearly more evident in response to 4000 Hz toneburst than 500 Hz and clicks.

**Table 1:** Incidence of the presence of CM for three stimuli, i.e., clicks, 500 Hz toneburst, and 4000 Hz toneburst, in Group A (individuals with ANSD) and Group B (individuals with normal hearing). CM: cochlear microphonics; ANSD: auditory neuropathy spectrum disorder.

Group	Clicks	500 Hz toneburst	4000 Hz toneburst
Group A (N = 46 ears)	47.8%	73.9%	73.9%
Group B (N= 24 ears)	0.04%	0.12%	62.5%

**Table 2:** The mean and standard deviation for the duration of cochlear microphonics in ms for three stimuli, i.e., clicks, 500 Hz toneburst, and 4000 Hz toneburst, in Group A.

Stimulus	Number of ears	Mean	SD
Clicks	22	3.05	1.50
500 Hz	34	7.50	2.42
4000 Hz	34	1.32	0.73

**Table 3:** The mean and standard deviation for the duration of cochlear microphonics in ms for three stimuli, i.e., clicks, 500 Hz toneburst, and 4000 Hz toneburst, in Group B.

Stimulus	Number of ears	Mean	SD
Clicks	1	1.07	-
500 Hz	3	0.58	0.57
4000 Hz	15	0.43	0.22

To statistically analyze the difference in duration between the types of stimuli used, in Group A, normality testing was done using the Shapiro-Wilk test, which showed that the data were not normally distributed. Thus, non-parametric tests were used. The results revealed that for all three stimuli used, there was a significant difference in the duration of CM on the Kruskal-Wallis test with p < 0.05. In addition, the Mann-Whitney U test revealed significant differences between clicks and 4000 Hz, clicks and 500 Hz, and 500 Hz and 4000 Hz. The number of ears with CM present in Group B was very less; hence, statistical analysis was limited to descriptive statistics.

## Discussion

The study revealed long ringing CM in individuals with ANSD as compared to individuals with normal hearing, which is similar to the findings of Soares et al. [12]. The absence of ABR peaks in ANSD with the presence of OAEs confirms the dysfunction of the afferent fibers but the increased duration of the CM implies increased activity of the outer hair cells. Since the efferent system controls the outer hair cells' activity and its function is compromised in ANSD, it may be concluded that the efferent system could possibly also be responsible for the increased duration of the CM. On the other hand, individuals with normal hearing having a normal efferent system would have lesser ringing CM. This is also supported by Santarelli et al. [7]. In addition, an increase in the amplitude of the CM has been reported in the literature [8] due to impaired hair cell metabolism as a result of pharmacological agents such as acetylsalicylic acid. Thus, it could also imply that the longer duration of CM at least in some individuals with ANSD could probably be attributed to a metabolic disorder leading to enhanced CM and impairment of the auditory nerve.

Clicks and tonebursts yielded different results in both groups in terms of the incidence of CM. There were ear differences even in the duration of ringing CM. Group A had observable CM in response to all three types of stimuli (clicks, 500 Hz tonebursts, and 4000 Hz tonebursts) but in Group B, none of the ears had CM in all three stimuli together. The presence of CM was highest in response to toneburst stimuli of both frequencies, followed by clicks in Group A. A similar trend in the incidence of CM is also seen in Group B. Likewise, the duration of ringing CM was also longer in response to low-frequency tonebursts than in response to clicks or high-frequency tonebursts. This is attributed to the duration of the stimuli [9]. This suggests that compared to clicks, toneburst stimuli are more sensitive to identifying ANSD, as there are chances to miss the CM if we restrict the diagnostic ABR protocol to clicks. A longer CM is more evident in response to 500 Hz toneburst stimuli (7.50 ms) followed by clicks and 4 kHz and is statistically significant. The number of cycles of CM is reported to be more when longer duration stimulus is used [9]. Basilar membrane ringing is more in response to 500 Hz toneburst, which is a long-duration stimulus of 10 ms, as compared to 4000 Hz, which is 1.25 ms, or clicks, which is 100 ms. Thus, the number of cycles will be more in response to 500 Hz as evidenced in the current study. However, as reported by Zhang [9], 4 kHz toneburst is longer in duration than clicks, and hence the number of cycles of CM must be more as compared to click-evoked CM. However, in this study, the number of cycles of CM has not been examined, and only the duration till which the CM exists on the time window has been explored. This could be the reason why the duration of CM in response to clicks is longer than the 4 kHz toneburst. Although the number of cycles has not been measured, the duration of CM (7.50 ms) is directly related to the number of cycles in the case of 500 Hz toneburst stimuli. As CM mimics the stimulus, the wavelength of each cycle is smaller for 4000 Hz, making it difficult to count the number of cycles, and even though the number of cycles might be more in response to 4 kHz than clicks, it may not be reflected in the duration of the CM (Table 2).

The difference between click ABR versus toneburst ABR could also be due to graded contraction of the middle ear muscles that causes selective enhancement of CM to certain tonal stimuli as cited by Starr et al. [8]. Thus, these contractions of muscles allow certain tonal frequencies to be transmitted through the middle ear. However, in the case of ANSD, this theory is not applicable. The muscle contraction in ANSD is possible to a variety of non-acoustic stimuli but typically, the acoustic reflexes are absent [8]. Thus, this explanation may not suffice for the differences in the duration of CM.

Limitations of the study

The study was limited to retrospective analysis of the data and hence the duration of CM was calculated manually using a scaling method as mentioned. Another drawback of the study was that the number of cycles of ringing of CM in response to the stimuli was not examined and only the duration of the ringing on the time window was looked into. Analyzing a larger data pool can also throw some insights and relating it to the presence and disappearance of OAEs may be suggested as future implications.

## Conclusions

Though both the groups have normal cochlear function as apparent from the distortion product otoacoustic emission findings, the difference in the properties of the CM suggests that these cochlear potentials are further controlled by some other physiological mechanisms. It is very evident from the study that a 500 Hz tone burst is more sensitive in identifying CM in ANSD followed by 4 kHz stimuli than clicks using scalp-recorded ABR. A time window of 15 ms or longer may be opted while using toneburst in cases when ANSD is suspected as CM lasts longer in duration. Thus, a 500 Hz toneburst must be included in the ABR protocol to avoid misdiagnosis as profound hearing loss, especially in cases where OAEs are absent or not recordable due to middle ear dysfunction. The use of click stimulus alone may obscure the CM. In addition, CM in normal hearing cochlea is less shown in scalp recording ABRs than in ECochG.
